# A *SMARTT *future for sports medicine

**DOI:** 10.1186/1758-2555-1-1

**Published:** 2009-01-19

**Authors:** Kai-Ming Chan, Masahiro Kurosaka

**Affiliations:** 1 Department of Orthopaedics and Traumatology, Prince of Wales Hospital, Faculty of Medicine, The Chinese University of Hong Kong, Hong Kong, PR China; 2 Department of Orthopaedic Surgery, Kobe University Graduate School of Medicine, Kobe, Japan

*Sports Medicine, Arthroscopy, Rehabilitation, Therapy & Technology (SMARTT) *is an open access, peer-reviewed, online journal published by BioMed Central. This new journal encourages and facilitates academic exchanges from different cultural, social, economical and ethnic perspectives, allowing unique research in fields related to the journal to be shared around the world.

With an expert Editorial team [1], we shall present quality research that reaches a wide readership amongst professionals working in different disciplines. By bringing together research from the fields of sports medicine, arthroscopy, rehabilitation, therapy and technology, which may previously have been found in a range of journals, we hope to provide a single home for the cross-fertilization of ideas. We also aim to promote collaboration amongst academic institutions, industrial and corporate research facilities to support integration for new technology and clinical applications.

*SMARTT *is the official journal of the Asia Pacific Orthopaedic Society for Sports Medicine (APOSSM) and is affiliated with the Japanese Orthopaedic Society for Knee Surgery Arthroscopy And Sports Medicine (JOSKAS) and five other societies. We hope that these strong links and support from authors will help to inspire clinicians, practitioners, scientists and engineers to work towards a common goal to improve the quality of life in the international community.

In order to cover all of the essential topics in disciplines related to the journal, *SMARTT *will consider manuscripts on sports science, exercise physiology, sports nutrition, sports psychology, sports medicine, arthroscopic surgery, sports, orthopaedic and computational biomechanics, manual therapy, physical therapy, musculoskeletal rehabilitation, biomedical devices and technology and sports equipment technology. The *SMARTT *'About' page [2] contains further information regarding the journal scope.

Timely publication and high visibility are two important features of this journal that makes it different from other traditional journals. Being an open access journal ensures rapid and efficient communication of research findings for the benefits of science, medicine and the general public. Authors can be assured their work is disseminated to the widest possible audience. The copyright belongs to the authors and they grant anyone the right to reproduce and disseminate the article on condition that the article has been correctly cited and no errors have been introduced [3].

Articles submitted to *SMARTT *will be published quickly and continuously online once accepted after the peer-review process, avoiding the delays commonly found in traditional journals in which submitted and accepted papers may have to wait for publication until enough articles have been collected for a particular issue. Three reviewers will be assigned for each submission, and reviewers will have up to three weeks to review the submitted article. After the receipt of the recommendation from the reviewers, the decision on whether to accept, reject or request major/minor revisions will be made by Co-Editors-in-Chief. The review time will be monitored by the Editorial Board to make it reasonably short for this journal.

We hope that you will join us in supporting this new journal, making it the home for high-quality research in the fields of sports medicine, arthroscopy, rehabilitation, therapy and technology.
